# Comparative Effects of *Escherichia coli* vs. *Porphyromonas gingivalis* Lipopolysaccharides on Osteogenic Differentiation and the Expression of lncRNAs in Periodontal Ligament Stem Cells

**DOI:** 10.3390/ijms27115006

**Published:** 2026-06-01

**Authors:** Tudor-Sergiu Suciu, Simion Bran, Ioana Berindan-Neagoe, Lajos Raduly, Oana Zanoaga, Livia Budisan, Andreea Nutu, Olga Soritau, Stefan Strilciuc, Daniel Leucuța, Dana Feștilă, Oana Almășan, Alexandra Iulia Aghiorghiesei, Mihaela Băciuț

**Affiliations:** 1 Department of Orthodontics and Dentofacial Orthopedics, “Iuliu Hațieganu” University of Medicine and Pharmacy, 400083 Cluj-Napoca, Romania; suciu.tudor.sergiu@elearn.umfcluj.ro (T.-S.S.);; 2 Department of Maxillofacial Surgery and Implantology, “Iuliu Hațieganu” University of Medicine and Pharmacy, 400029 Cluj-Napoca, Romania; 3 Department of Genomics, “Iuliu Hațieganu” University of Medicine and Pharmacy, 400337 Cluj-Napoca, Romaniaolgasoritau@yahoo.com (O.S.);; 4 Doctoral School, “Iuliu Hațieganu” University of Medicine and Pharmacy, 400029 Cluj-Napoca, Romania; 5 Academy of Medical Sciences, 030167 Bucharest, Romania; 6 Department of Medical Informatics and Biostatistics, “Iuliu Hațieganu” University of Medicine and Pharmacy, 400349 Cluj-Napoca, Romania; 7 Department of Prosthetics and Dental Materials, “Iuliu Hațieganu” University of Medicine and Pharmacy, 400006 Cluj-Napoca, Romania; oana.almasan@umfcluj.ro (O.A.);

**Keywords:** periodontal inflammation, periodontal ligament stem cells, osteogenic differentiation, lipopolysaccharide, long non-coding RNAs, *Porphyromonas gingivalis*

## Abstract

Periodontal ligament mesenchymal stem cells (PL-MSCs) are vital for both periodontal regeneration and alveolar bone maintenance, including their turnover during orthodontic therapy. Chronic periodontal inflammation, mainly caused by Gram-negative bacterial lipopolysaccharides (LPS), interferes with osteogenic differentiation and leads to bone loss. Increasing evidence indicates that long non-coding RNAs (lncRNAs) link inflammatory signaling to osteogenic regulation, but their specific role in LPS-driven modulation of PL-MSC osteogenesis is not well understood. The aim of this study was to assess the effects of LPS from two bacterial strains on PL-MSCs differentiation. Human PL-MSCs were cultured under standard stem cell or osteogenic conditions and treated with LPS from *Escherichia coli* or *Porphyromonas gingivalis*. Mineralization was assessed using Alizarin Red staining. Osteogenic differentiation was evaluated through immunocytochemical analysis of osteopontin, collagen type 1, osteocalcin, osteonectin, and dentin matrix protein-1 (DMP-1). Expression levels of lncRNAs growth arrest-specific transcript 5 (*GAS5*), Metastasis-Associated Lung Adenocarcinoma Transcript 1 (*MALAT1*), maternally expressed gene 3 (*MEG3*) and Nuclear Enriched Abundant Transcript 1 (*NEAT1*) were measured by real-time PCR at 6, 24 and 48 h of LPS exposure. Exposure to *E. coli* LPS significantly inhibited extracellular matrix mineralization and decreased the expression of key osteogenic markers, indicating impaired osteoblast maturation. In contrast, *P. gingivalis* LPS caused a partial, dysregulated osteogenic response, marked by increased expression of osteopontin, osteonectin, and dentin matrix protein-1 (DMP-1), but without complete differentiation. LPS types altered lncRNA expression profiles, suggesting that non-coding regulatory networks are involved in inflammation-induced osteogenic dysregulation. Multivariate analyses showed decreased expression of *GAS5*, *MEG3*, and *MALAT1* in the LPS vs. CTR comparison, decreased COL1A1 in LPS-PG vs. CTR, and increased OSTEOPONTIN in LPS vs. CTR. Differentiation was significantly associated with reduced expression of *XIST* and *NEAT1*. Time exerted significant effects on *GAS5*, *MEG3*, *XIST*, and *MALAT1*, with lower expression at 48 h compared with 6 h, and on COL1A1, which was significantly reduced at both 24 h and 48 h relative to 6 h. Bacterial LPS disrupt osteogenic differentiation of PL-MSCs depending on the species, affecting matrix formation, mineralization, and lncRNA expression. These findings highlight lncRNA-mediated communication between inflammatory signals and osteogenic pathways, providing new insights into the molecular mechanisms of inflammation-related bone remodeling in periodontal disease and orthodontic movements. Targeting lncRNA-regulated pathways could be a promising strategy to enhance periodontal regeneration during inflammation and also ensure optimum outcomes in orthodontic therapy.

## 1. Introduction

Periodontal ligament stem cells (PL-MSCs) are highly specialized mesenchymal stem cells vital for maintaining periodontal health, regeneration, and repair. Due to their neural crest origin and close association with alveolar bone and cementum, PL-MSCs have a strong potential for osteogenic and odontogenic differentiation, making them an important model for studying bone formation and regeneration in both healthy and diseased conditions [1]. PL-MSCs meet the minimum criteria for phenotypic markers and multipotential differentiation. PL-MSCs are positive for adult mesenchymal markers CD105, CD73, and CD90 and negative for CD34, CD45, CD11a, CD19, and HLA-DR and can differentiate into osteocytes, adipocytes, and chondrocytes in vitro [2,3] or during orthodontic therapy, respectively. In addition to their regenerative functions, PL-MSCs actively regulate the immune response and adjust their behavior in response to inflammatory signals within the periodontal microenvironment [2].

Periodontitis is a chronic inflammatory disease that compromises the integrity of the tooth-supporting tissues, that is the gingiva, periodontal ligament, and alveolar bone, collectively known as the periodontium. Accordingly, dysbiosis by itself may not necessarily precipitate periodontitis, but it could initiate disease in the context of other risk factors associated with host genotype, stress, diet, or risk-related behavior such as smoking [3]. For instance, there might be individuals who can tolerate dysbiosis by virtue of their intrinsic immunoinflammatory status; hyporesponsive or loss-of-function polymorphisms in immune response genes could attenuate inflammation and prevent the development of overt disease. Bacterial dysbiosis will only lead to disease in susceptible hosts, as some individuals remain periodontally healthy despite massive tooth-associated biofilm formation, whereas others with less biofilm accumulation are extremely susceptible to periodontitis [1].

Chronic periodontal disease involves ongoing inflammation caused by Gram-negative bacteria, leading to the gradual breakdown of periodontal tissues and loss of alveolar bone. The etiology of periodontitis is complex, acting at multiple levels: (1) the presence of dysbiotic microbial populations with the induction of inflammation; (2) genetic factors at the host level [2]; and environmental factors and systemic health status [4]. Lipopolysaccharides (LPS), the main components of the outer membranes of Gram-negative bacteria such as *Escherichia coli* and *Porphyromonas gingivalis*, are key mediators of periodontal inflammation [5]. LPS primarily exerts its effects through Toll-like receptor signaling, affecting cell survival, differentiation, cytokine production, and extracellular matrix remodeling [6]. Studies have shown that bacterial LPS can significantly affect osteoblast function, inhibit mineralization, and disrupt bone homeostasis, although these effects may vary depending on the species and the differentiation stage of the cells involved. Osteogenic differentiation of mesenchymal stem cells is a carefully regulated process involving the coordinated expression of extracellular matrix proteins, including collagen type 1, osteopontin, osteocalcin, osteonectin, and dentin matrix protein-1 (DMP-1). These markers reflect different stages of osteoblast maturation and mineralization [7,8]. Inflammatory stimuli, such as LPS, have been shown to influence the expression of these proteins, thereby affecting matrix organization and mineralization capacity. However, detailed data on the specific effects of LPS from periodontal versus non-periodontal bacteria on PL-MSC osteogenesis are still limited.

In recent years, with the discovery of the non-coding genome, previously considered a “garbage” genome, long non-coding RNAs (lncRNAs) have emerged as key regulators of cellular states, stem cell fate, osteogenic differentiation, and inflammatory responses. LncRNAs are non-coding transcripts longer than 200nt sequences, involving RNA polymerase I (Pol I), Pol II, and Pol III transcribed RNAs and RNAs from processed introns [9,10]. Among them, several lncRNAs, including growth arrest-specific transcript 5 (*GAS5*), Metastasis Associated Lung Adenocarcinoma Transcript 1 (*MALAT1*), maternally expressed gene 3 (*MEG3*), and Nuclear Enriched Abundant Transcript 1 (*NEAT1*), regulate osteoblast differentiation, apoptosis, and immune signaling pathways. Moreover, long non-coding RNAs (lncRNAs) serve as regulators in the growth of cellular organisms, including bone formation, and are involved in the balance between osteoblastogenesis and osteoclastogenesis [11,12]. Importantly, these lncRNAs also respond to inflammatory stimuli and might act as molecular links connecting inflammation to impaired bone formation [12,13]. Despite increasing evidence of their role in bone biology, the specific contribution of lncRNAs to LPS-mediated modulation of PL-MSC osteogenesis remains poorly understood [14].

The aim of the present study was to investigate the effects of LPS derived from *E. coli* and *P. gingivalis* on the osteogenic differentiation of periodontal ligament mesenchymal stem cells. We evaluated mineralization capacity, expression of key osteogenic markers, and the transcriptional regulation of selected lncRNAs under inflammatory conditions. Understanding the molecular mechanisms by which bacterial components influence PL-MSC differentiation may provide new insights into pathogenesis of periodontium remodeling and identify potential therapeutic targets for regenerative strategies for orthodontic therapies and many more dental medicine domains.

## 2. Results

### 2.1. Immunophenotypic Characterization of Cells Isolated from the Periodontal Ligament

#### Mineralization Evaluation by Alizarin Red Staining

**Figure 1:**
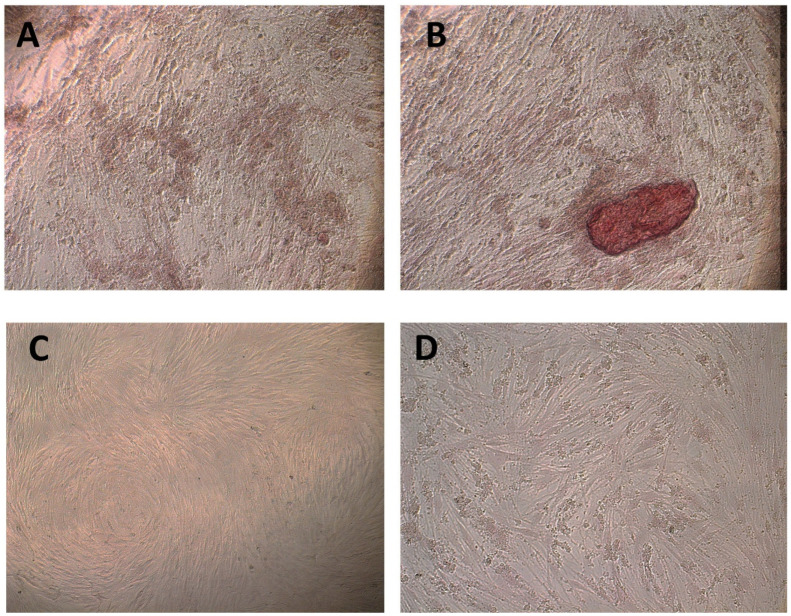
Alizarin red staining of pre-differentiated PL-MSC cells into osteoblasts. (**A**) Control PL-MSCs cultivated in standard stem cell medium; (**B**) control PL-MSCs induced to differentiate into osteoblasts with osteogenic medium; (**C**) pre-differentiated PL-MSCs cultivated in standard medium after 5 days of treatment with a single dose of 100 ng/mL LPS*_E. coli_*; (**D**) pre-differentiated PL-MSCs cultivated in osteogenic medium after 5 days of treatment with a single dose of 100 ng/mL LPS*_E. coli_*.

In Figure 1, it is observed that pre-osteoblastic cells that were treated with a single dose (100 ng/mL) of LPS*_E. coli_* and then cultured for an additional 5 days in osteogenic or standard stem cell medium had a much lower mineralization rate than control cells, with no calcium deposits in the extracellular environment. A similar finding was also reported by Guo et al. (2014) [15].

### 2.2. Evaluation of Osteogenesis by Immunocytochemistry

**Figure 2:**
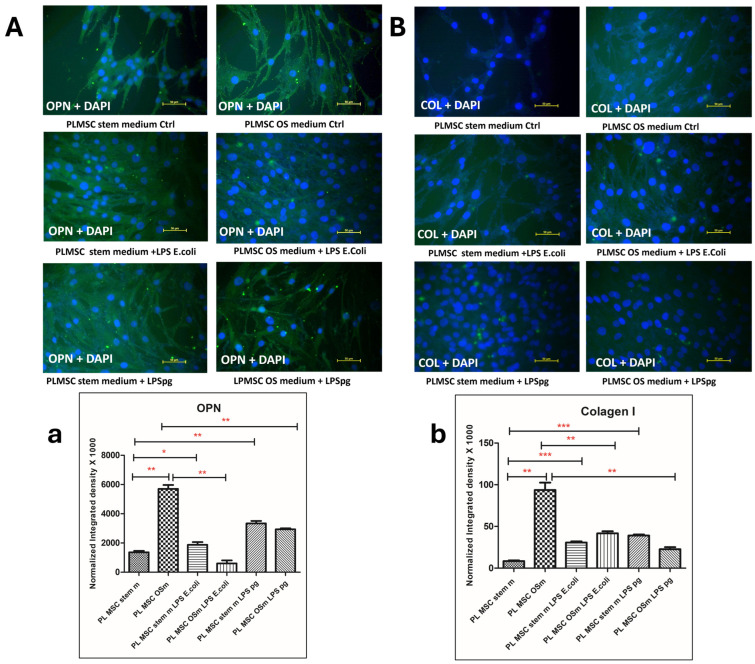
Immunocytochemical analysis of osteopontin and collagen 1a expression in PL-MSC following LPS*_E. coli_* and LPS*_Pg_* treatment. (**A**) Osteopontin (FITC) expression of control PL-MSC cells and PL-MSC cells treated with 1 µg/mL LPS*_E. coli_* and 1 µg/mL LPS*_Pg_* (magnification objective ×40); (**B**) collagen 1a (FITC) expression of control PL-MSC cells and PL-MSC cells treated with 1 µg/mL LPS*_E. coli_* and 1 µg/mL LPS*_Pg_* (magnification objective ×40). The scale bar was set to 50 μm. (**a**,**b**) represent measurements of fluorescence intensity as normalized integrated density using ImageJ software. The graphs and statistical analysis were performed with GraphPad Prism 5 using a two-paired *t*-test, with settings of statistical significance at *p* ≤ 0.05, with statistical significance noted as * *p* < 0.05, ** *p* < 0.01 and *** *p* < 0.001.

Periodontal ligament stem cells (PL-MSC) cultured in standard stem cell medium and osteogenic medium (OS) were treated with a single dose of LPS*_E. coli_* and LPS*_Pg_* at a concentration of 1 µg/mL and further cultivated for 5 days. Immunocytochemical staining for osteopontin FITC expression was performed, with nuclei counterstained with DAPI. Weak OPN expression was also observed in undifferentiated cells. OS medium induced OPN expression on control cells. LPS from *E. coli* decreases OPN expression in both stem and OS medium, and LPS*_Pg_* induces an increase in OPN expression, especially in OS medium (Figure 2).

In Figure 2, staining with the collagen 1a antibody is illustrated. It is observed that undifferentiated cells do not yet express collagen after 5 days of culture in standard stem medium, and OS medium induces very weak Coll 1A expression in control cells. LPS*_E. coli_* induces a weak expression of collagen 1A intracellularly in standard stem medium and in the extracellular space in OS medium. LPS*_Pg_* induces a weak increase in extracellular collagen 1A expression in standard stem medium.

**Figure 3:**
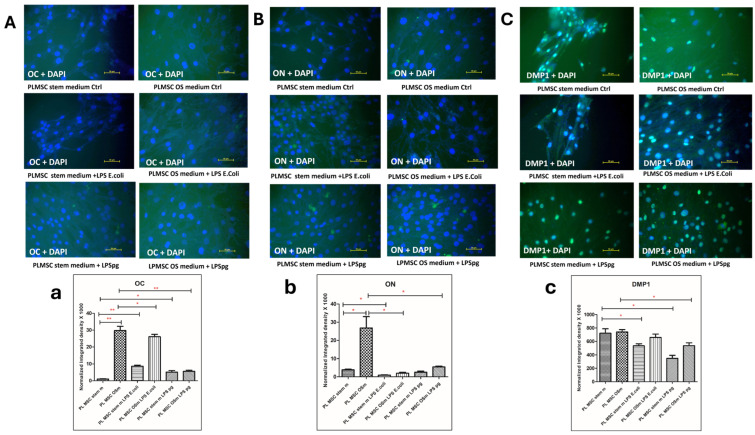
Immunocytochemical analysis of osteocalcin, osteonectin, and DMP-1 expression in PL-MSC following LPS*_E. coli_* and LPS*_Pg_* treatment. (**A**) Osteocalcin (FITC) expression of control PL-MSC cells and PL-MSC cells treated with 1 µg/mL LPS*_E. coli_* and 1 µg/mL LPS*_Pg_* (magnification objective ×40); (**B**) osteonectin (FITC) expression of control PL-MSC cells and PL-MSC cells treated with 1 µg/mL LPS*_E. coli_* and 1 µg/mL LPS*_Pg_* (magnification objective ×40); (**C**) dentin matrix protein-1 (DMP-1) (FITC) expression of control PL-MSC cells and PL-MSC cells treated with 1 µg/mL LPS*_E. coli_* and 1 µg/mL LPS*_Pg_* (magnification objective ×40). The scale bar was set to 50 μm. (**a**–**c**) represent measurements of fluorescence intensity as normalized integrated density using ImageJ software. The graphs and statistical analysis were performed with GraphPad Prism 5 using a two-paired *t*-test, with settings of statistical significance at *p* ≤ 0.05 with statistical significance noted as * *p* < 0.05, ** *p* < 0.01.

Undifferentiated PL-MSC cells do not express osteocalcin (OC) after 5 days, nor do cells pre-differentiated with OS medium after 5 days. LPS*_E. coli_* and LPS*_Pg_* do not induce OC expression after 5 days of culture (Figure 3).

Undifferentiated PL-MSC cells did not express osteonectin (ON), and cells pre-differentiated in OS medium expressed it very weakly after 5 days of culture. Treatment with LPS*_E. coli_* induced weak expression in the presence of standard stem medium. LPS*_Pg_* determined ON expression in standard stem medium but also in OS medium (weak expression) (Figure 3).

Another protein studied is DMP-1, which is highly expressed in odontoblasts and osteoblasts/osteocytes. DMP-1 plays an essential role in tooth and bone mineralization as well as phosphate homeostasis. Besides its function in the extracellular matrix, it can also enter the nucleus and act as a transcription factor. It is also expressed by adult MSCs [16,17]. In our study, undifferentiated cells showed high levels of DMP-1 expression both in the nucleus and in the cytoplasm. Cells pre-differentiated in OS medium exhibited less intranuclear DMP-1, but it seems to be secreted into the extracellular space. LPS from *E. coli* induces nuclear and intracytoplasmic DMP-1 expression, whereas cultivation in OS medium promotes extracellular export of the protein. LPS*_Pg_* induces DMP-1 expression in the nucleus and intracellularly after five days of treatment in standard stem medium. The most intense expression occurs in the nuclei with subsequent appearance in the extracellular space (Figure 3).

### 2.3. Gene Expression Evaluation Under Different Conditions

The expression of genes involved in the osteogenesis process was analyzed at different time intervals (6, 24 and 48 h) in different PL-MSC cell cultivation conditions: standard medium (CTRL) and osteogenic differentiation medium (OS). Treatment with LPS*_E. coli_* and LPS*_Pg_* was performed at doses of 1 µg/mL.

Osteopontin expression displayed a distinct response to bacterial LPS stimulation. In control osteogenic conditions, osteopontin expression was detected at moderate levels, consistent with its role as an early-to-intermediate osteogenic and matrix-remodeling marker.

Analysis of collagen 1 expression revealed a general reduction in collagen type 1 transcription under inflammatory conditions compared to osteogenic controls. In control cultures, collagen 1 expression was maintained, consistent with its role as a major structural component of the extracellular matrix during early osteogenic differentiation.

Expression of osteocalcin, a late marker of osteoblast maturation, was low or undetectable across all experimental conditions at the examined time point. Control osteogenic cultures did not show a significant increase in osteocalcin expression, indicating that terminal osteogenic differentiation had not yet been reached.

### 2.4. Differential Expression for lncRNAs in Different Conditions

Quantitative RT-PCR analysis of *GAS5* expression was conducted in periodontal ligament mesenchymal stem cells (PL-MSCs) cultured in standard stem cell medium or osteogenic medium and treated with lipopolysaccharides (LPS) from *Escherichia coli* (1 µg/mL) or *Porphyromonas gingivalis* (1 µg/mL) for 6, 24, and 48 h. Gene expression levels were normalized to the housekeeping genes *GAPDH* and *B2M* and calculated using the ΔΔCt method. Data are presented as mean ± SD. Statistically significant differences compared to untreated control cells are indicated (*p* < 0.05).

Relative *MALAT1* expression levels in periodontal ligament mesenchymal stem cells cultured in stem cell medium or osteogenic medium and exposed to *E. coli* LPS (1 µg/mL) or *P. gingivalis* LPS (1 µg/mL) for 6, 24, and 48 h. Expression values were normalized to *GAPDH* and *B2M* and expressed relative to untreated control samples. Results represent mean ± SD, with statistical significance determined by Student’s *t*-test (*p* < 0.05).

The expression level of the *MEG3* lncRNA was measured in standard/osteogenic medium in periodontal ligament mesenchymal stem cells, which were treated with lipopolysaccharides from *E. coli* (1 µg/mL) or *P. gingivalis* (1 µg/mL) for 6, 24 and 48 h. Data are presented as mean ± SD. Significant differences compared to untreated controls are indicated (*p* < 0.05).

Relative *NEAT1* expression was assessed in PL-MSCs cultured in stem cell or osteogenic medium and treated with lipopolysaccharides from *E. coli* (1 µg/mL) or *P. gingivalis* (1 µg/mL) for 6, 24, and 48 h. Gene expression was normalized to *GAPDH* and *B2M* and expressed as fold change versus untreated control cells. Values are presented as mean ± SD, with statistical significance assessed by Student’s *t*-test (*p* < 0.05).

**Figure 4:**
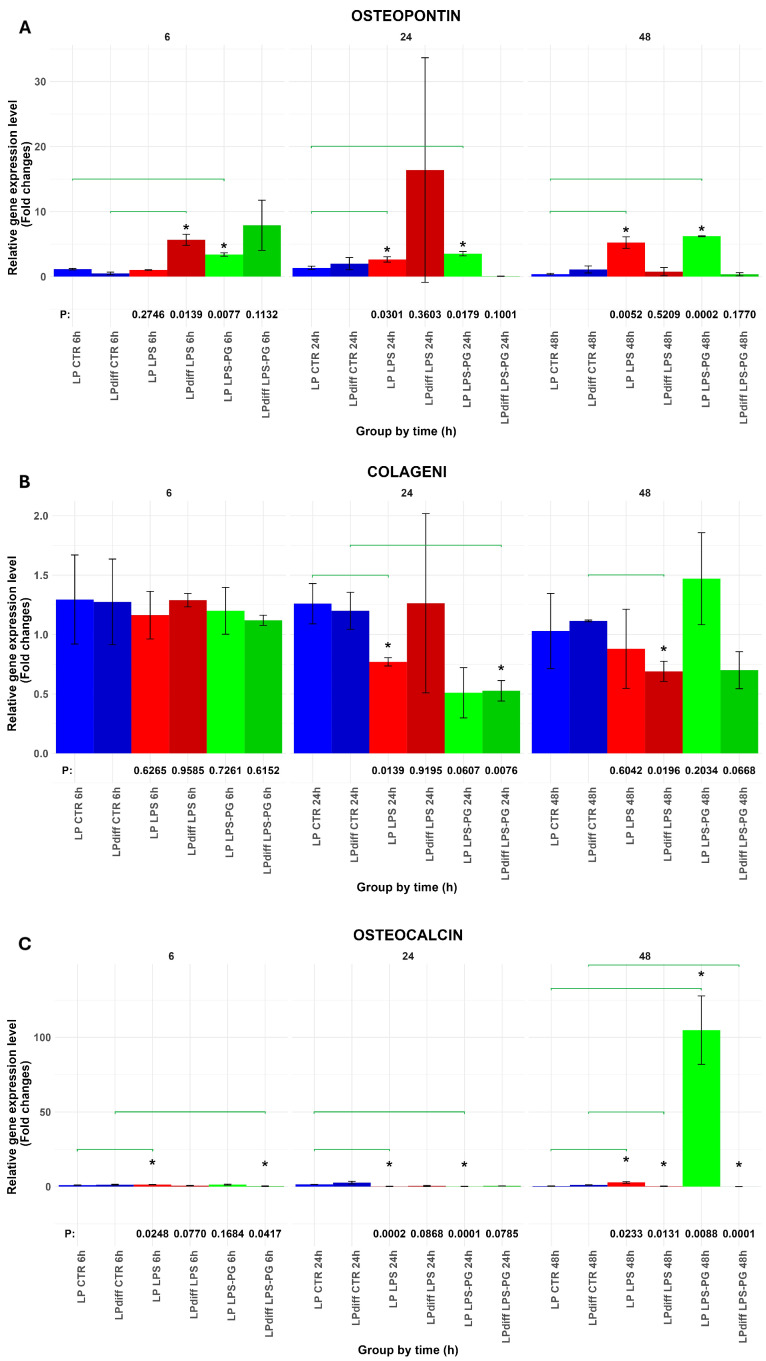
Time-dependent regulation of osteogenic gene expression in PL-MSC under LPS stimulation. Data are presented as mean ± standard deviation. (**A**) Time-dependent regulation of osteopontin expression in periodontal ligament mesenchymal stem cells following LPS stimulation. PL-MSC CTRL cells cultivated with standard stem cell medium; PL-MSC OS cells cultivated with osteogenic medium (OS); PL-MSC CTRL LPS*_E._ _coli_*; PL-MSC OS LPS*_E. coli_*; PL-MSC CTRL LPS*_Pg_*; PL-MSC OS LPS*_Pg_*. (**B**) Time-dependent regulation of collagen type 1 (COL1A1) expression in periodontal ligament mesenchymal stem cells following LPS stimulation. (**C**) Time-dependent modulation of osteocalcin expression by bacterial lipopolysaccharides. *p*-values from tests comparing each sample with the corresponding control group within the same culture condition; * and green lines, statistically significant differences (*p* < 0.05).

**Figure 5:**
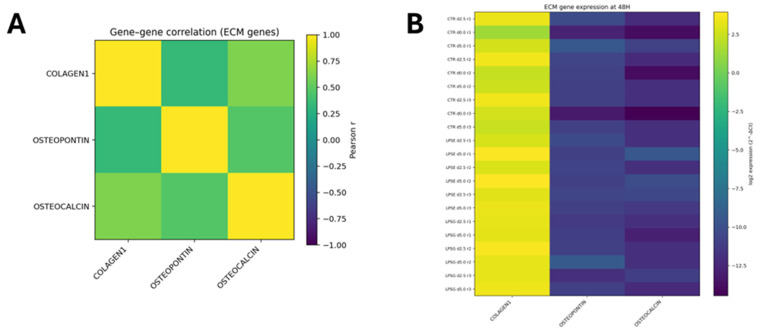
Gene–gene correlation and expression profiling of extracellular-matrix-associated transcripts in PL-MSCs at 48 h. (**A**) Gene–gene correlation analysis of ECM-associated transcripts using Pearson correlation coefficients between collagen 1, osteopontin, and osteocalcin across all samples at 48 h. (**B**) Heatmap of extracellular-matrix-related gene expression at 48: collagen 1, osteopontin, and osteocalcin at 48 h.

**Figure 6:**
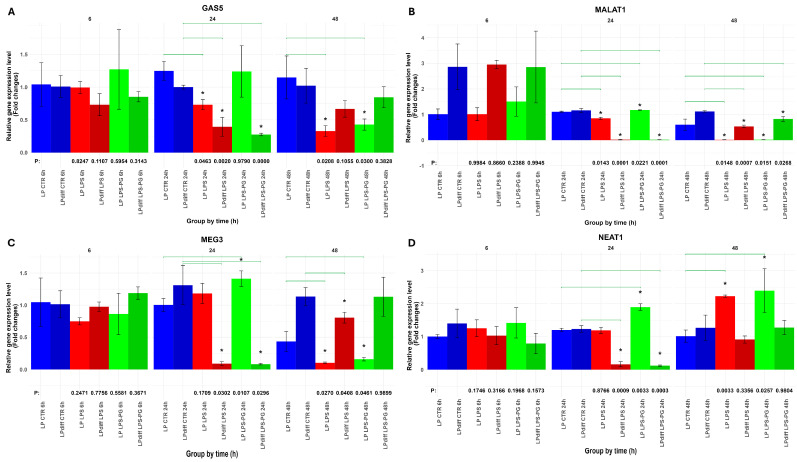
Time-dependent expression profiles of lncRNAs *GAS5*, *MALAT1*, *MEG3*, and *NEAT1* in PL-MSC under inflammatory and osteogenic conditions. Data are presented as mean ± standard deviation. (**A**) Relative expression of *GAS5* in periodontal ligament mesenchymal stem cells under inflammatory and osteogenic conditions. (**B**) Modulation of *MALAT1* expression in PL-MSCs following exposure to bacterial lipopolysaccharides. (**C**) *MEG3* expression profile in PL-MSCs during osteogenic differentiation under inflammatory conditions. (**D**) *NEAT1* expression in PL-MSCs exposed to inflammatory stimuli. *p*-values from tests comparing each sample with the corresponding control group within the same culture condition; * and green lines, statistically significant differences (*p* < 0.05).

**Figure 7:**
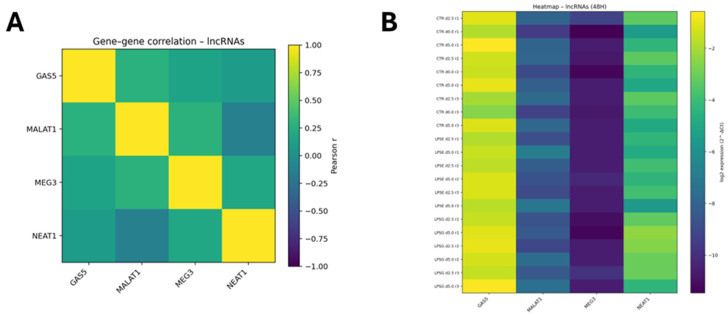
Gene–gene correlation and expression profiling of lncRNAs at 48 h. (**A**) Gene–gene correlation analysis of lncRNAs at 48 h. Pearson correlation coefficients were calculated between *GAS5*, *MALAT1*, *MEG3*, and *NEAT1* across all samples. (**B**) Heatmap of lncRNA expression at 48 h showing normalized expression of the long non-coding RNAs *GAS5*, *MALAT1*, *MEG3*, and *NEAT1*; qPCR data were normalized to the mean Ct of GAPDH and B2M, and values are displayed as log_2_.

**Figure 8:**
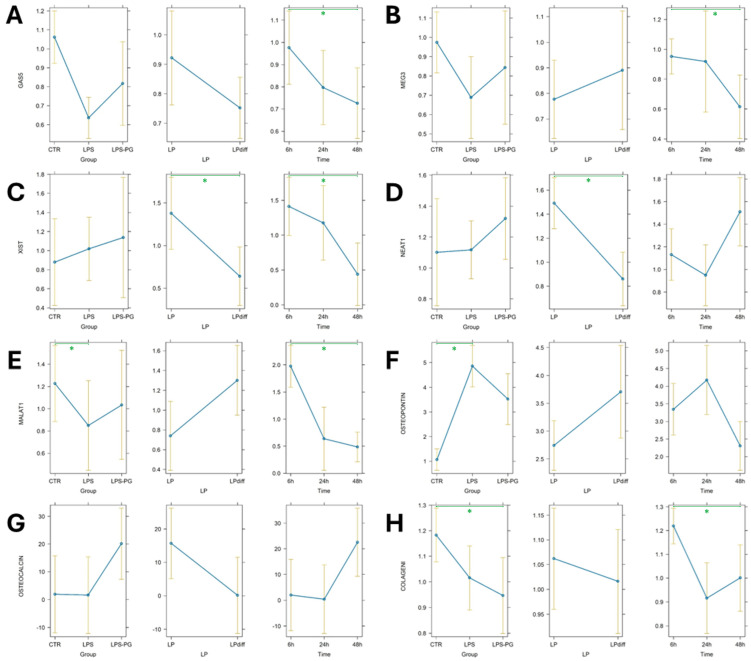
Time-dependent effect plots of gene expression in PLMSC under different groups and differentiation conditions. Data are presented as coefficient ± confidence interval. (**A**–**E**) Long non-coding RNAs (lncRNAs): *GAS5*, *MEG3*, *XIST*, *NEAT1*, *MALAT1*. (**F**–**H**) Osteogenic markers: osteopontin (OPN), osteocalcin (OC), and collagen type 1 (COL1A1). Models include robust estimations, and transformed data were applied where indicated. * and green lines, statistically significant differences (*p* < 0.05). Abbreviations: PL-MSC, periodontal ligament mesenchymal stem cells; lncRNA, long non-coding RNA.

To isolate the independent effects of group, differentiation, and time on gene activity, multiple linear regression models were fit and are presented in Table 1 and Figure 4, Figure 5, Figure 6, Figure 7 and Figure 8.

For all the genes studied, the Group variable was associated with statistically significant changes in expression in four genes. For *GAS5*, expression was decreased in both LPS vs. CTR and LPS-PG vs. CTR comparisons, with the former significant and the latter approaching significance. Other significant expression downregulations were observed for *MEG3* and *MALAT1* in the LPS vs. CTR comparison, and for COLLAGEN 1 in the LPS-PG vs. CTR comparison. A significant upregulation of OSTEOPONTIN expression was observed in the LPS vs. CTR comparison. No statistically significant changes in expression associated with the group were found for *XIST* and *NEAT1*, where slight increases were observed, and for OSTEOCALCIN, where slight decreases were observed.

The effect of cell differentiation on gene expression depends on the gene of interest. For six of the assessed genes (*GAS5*, *XIST*, *NEAT1*, Osteopontin, Osteocalcin, and Collagen 1), the differentiation was associated with lower gene expression, although only *XIST* and *NEAT1* were statistically significant, and *GAS5* and Osteocalcin were close to the level of significance. For the other two assessed genes (*MEG3* and *MALAT1*), differentiation was associated with higher gene expression, though only *MALAT1* approached significance.

The effect of time on gene expression varies by gene. Many of the assessed genes showed decreased gene expression over time, including *GAS5*, *MEG3*, *XIST*, and *MALAT1*, with the decrease more pronounced at 48 h than at 24 h. Significant differences in this respect were observed only between 48 h and 6 h. A few genes showed a slight decrease in gene expression at 24 h, followed by an increase at 48 h above that at 6 h for *NEAT1* and Osteocalcin; the effect was close to the significance level only for *NEAT1* when comparing gene expression at 48 h with that at 6 h. Collagen 1 gene expression decreased significantly at 24 h compared to 6 h, then slightly increased at 48 h without surpassing the 6 h level, and reached significance only in the last comparison. Osteopontin gene expression increased slightly at 24 h and then decreased at 48 h to levels below those at 6 h, but the changes did not reach significance.

## 3. Discussion

Periodontal disease, one of the most common causes of destruction of the periodontium and tooth loss, is triggered by chronic inflammation driven by bacterial colonization in dental plaque. In addition to local involvement, associations with various systemic diseases are increasingly being described, suggesting a role for chronic inflammation in their occurrence (coronary heart disease, atherosclerosis, dementia or other neurodegenerative diseases, and oral, lung, and pancreatic cancers) [18]. The Gram-negative bacterium *Porphyromonas gingivalis* (*P. gingivalis*) is the main causative agent of periodontitis and the subsequent inflammatory immune response [19]. It seems that the host response to bacterial infection plays an important role in periodontal bone resorption, with direct or indirect stimulation of osteoclastogenesis by molecules such as prostaglandins, leukotrienes, cytokines, and chemokines [20]. The hallmark of periodontitis is an increase in the concentrations of pro-inflammatory cytokines such as IL-1β and TNF, which directly induce bone loss by increasing the RANKL/osteoprotegerin (osteoclastogenesis inhibitory factor) ratio [21].

Periodontal ligament stem cells are resident mesenchymal progenitors within the periodontal ligament and are responsible for the regeneration of the damaged periodontal bone tissues by their osteogenic potential. Periodontal ligament stem cells are important due to their features of self-renewal, multipotency, and immunomodulation, which highlight their capability in periodontal complex regeneration but also in other dental and non-dental tissues [1,22].

PL-MSCs are affected in pro-inflammatory environments (TNF-α, IL-1, or other inflammatory mediators such as LPS), leading to inhibition of their osteoregenerative capacity. Wang et al. 2020 demonstrated that LPS inhibited the osteogenic capacity of PDLSCs by downregulating ephrinB2 via toll-like receptor 4 (TLR4), and the osteogenic potential was partially reversed by ephrinB2 overexpression in PDLSCs [23].

LPS*_Pg_* behaves differently from LPS*_E. coli_* by binding to TLR2 and inducing upregulation of TLR2 and can evade TLR4 stimulation of macrophages [24,25]. LPS*_Pg_* also induces DNA hypermethylation of RUNX2 in periodontal ligament fibroblasts, leading to decreased RUNX2 expression and impaired periodontal regeneration [26]. LPS*_Pg_* acts directly on osteoblasts or progenitors by reducing the number of functional cells and impairing their attachment, thereby hampering bone regeneration. Indirect effects are manifested by stimulation of osteoclastic cell activity via inflammatory cytokines, inducing bone resorption [27].

Multivariate analyses revealed significant, gene-specific independent effects of group, differentiation, and time on transcript levels. Group assessment was significantly associated with decreased expression of *GAS5*, *MEG3*, and *MALAT1* in the LPS vs. CTR comparison, decreased COL1A1 in LPS-PG vs. CTR, and increased OSTEOPONTIN in LPS vs. CTR. Differentiation was significantly associated with reduced expression of *XIST* and *NEAT1*. Time exerted significant effects on *GAS5*, *MEG3*, *XIST*, and *MALAT1*, with lower expression at 48 h compared with 6 h, and on COL1A1, which was significantly reduced at both 24 h and 48 h relative to 6 h. These results indicate that inflammatory stimulation, differentiation status, and exposure time selectively and independently modulate key lncRNAs and osteogenic markers.

Our study shows that bacterial lipopolysaccharides have unique and significant effects on the osteogenic differentiation of periodontal ligament mesenchymal stem cells, affecting both mineralization capacity and the expression of osteogenic markers, as well as newly identified ncRNAs, which are known to play regulatory roles. Our results support the idea that inflammatory stimuli from diverse bacterial sources influence PL-MSC behavior differently and impact bone remodeling by contributing to an imbalance between bone formation and resorption.

Alizarin red staining revealed a significant decrease in extracellular matrix mineralization after exposure to *E. coli*-derived LPS in pre-differentiated osteoblasts. Previous studies show similar results, indicating that LPS inhibits calcium deposition and impairs osteoblast maturation. This process activates pro-inflammatory signaling pathways and reduces the expression of osteogenic transcription factors. The absence of detectable calcium nodules suggests that LPS affects the later stages of osteogenic differentiation, a finding also reported by Guo et al., who observed a significant decrease in mRNA expression of osteoblast-related genes and alkaline phosphatase (ALP) activity in MC3T3-E1 cells, leading to inhibition of osteogenic differentiation via activation of the JNK pathway. Also, LPS induced osteoblast apoptosis by activating the caspase-3 pathway [15]. Likewise, LPS*_Pg_* extract (100 ng/mL) inhibited bone nodule development and ALP activity in primary fetal rat calvaria cells, without affecting cell proliferation and viability [28].

Immunocytochemistry revealed that LPS influences osteogenic marker expression in distinct ways. Lower levels of osteopontin were observed after *E. coli* exposure in both native and differentiated PL-MSCs, consistent with its role in inflammatory processes. Conversely, *P. gingivalis* LPS increased osteopontin levels, especially when cells were directed toward bone formation, indicating distinct signaling patterns across microbial types. Osteopontin functions during both bone development and tissue inflammation. When levels rise after periodontal exposure to LPS, it could indicate that the body is trying to repair itself—or that it is becoming imbalanced. Kadono et al. found that LPS*_Pg_* strongly inhibited osteocalcin mRNA expression in rat calvarial cells during the first 7 days and moderately inhibited osteopontin levels on day 14, with a further marked reduction on day 21 [28].

Collagen type 1, the primary structural component of the bone extracellular matrix, exhibited weak and delayed expression across different conditions, suggesting that early inflammatory exposure may impede proper matrix formation. The lack of osteocalcin expression further confirms that complete osteoblast differentiation was not achieved under inflammatory conditions, consistent with previous studies reporting delayed or suppressed osteocalcin production in LPS-treated cells. This limitation is further supported by the low or undetectable levels of osteocalcin, a late-stage marker of osteoblast maturation, suggesting that PL-MSCs did not reach terminal differentiation within the 48-h and 5-day timeframes. In addition, although multivariate analysis identified *GAS5*, *MALAT1*, *MEG3*, and *NEAT1* as key components associated with the inflammatory response, these findings remain correlative. Further functional studies, such as knockdown or overexpression approaches, are required to establish the precise regulatory roles of these lncRNAs in PL-MSC osteogenesis. These data are consistent with other studies showing that periodontal ligament stem cells (PDLSCs) are affected by LPS*_Pg_* exposure, with inhibition of ALP activity, COL1A, and osteocalcin production and mineralization. LPS*_Pg_* also induced the synthesis of IL-8β, IL-6, and IL-8 [29]. Osteonectin expression showed a similar pattern, with a slight increase in response to *P. gingivalis* LPS, indicating partial activation of osteogenic pathways without full maturation.

DMP-1 exhibited a distinct expression pattern, being highly expressed in undifferentiated PL-MSCs and dynamically redistributed between nuclear, cytoplasmic, and extracellular compartments upon osteogenic induction and LPS treatment. These findings are consistent with DMP-1’s multifunctional role as both an extracellular matrix protein and a transcriptional regulator. The enhanced nuclear and extracellular expression observed in response to *P. gingivalis* LPS may indicate an adaptive response aimed at maintaining mineral homeostasis under inflammatory stress.

Long non-coding RNAs have emerged as key regulators that connect inflammatory signals to stem cell fate decisions. In this study, changes in *GAS5*, *MALAT1*, *MEG3*, and *NEAT1* expression after LPS exposure indicate that these lncRNAs are involved in suppressing osteogenic differentiation during inflammation in PL-MSCs. lncRNAs have been shown to modulate bone morphogenetic protein (BMP), the WNT/β-catenin/RUNX2 pathway, and the transforming growth factor-β (TGF-β)/Smad3 pathway [30].

*GAS5*, a major lncRNA, has been shown to be altered in many inflammatory diseases. It is widely known as a negative regulator of cell proliferation and osteogenesis. *GAS5* is involved in the development and prognosis of some bone diseases such as osteoporosis and osteosarcoma [30]. It acts as a competing endogenous RNA (ceRNA), binding osteogenesis-promoting microRNAs and inhibiting pathways such as the Wnt/β-catenin pathway. The inflammatory process, which has been shown to increase *GAS5* levels under specific conditions, suppresses RUNX2 activity and hinders osteoblast differentiation, consistent with the reduced mineralization observed in our study [31]. Chronic activation of inflammatory cascades has been shown to interfere with RUNX2 activity, collagen production, and mineral deposition, ultimately contributing to bone loss and impaired tissue regeneration. Similar mechanisms have been described in inflammatory tumor microenvironments, where lncRNAs regulate the balance between inflammation and differentiation processes.

Another lncRNA well known for its role in inflammatory processes, *MALAT1*, plays a dual role in inflammation and osteogenesis [32,33,34]. It has been shown to enhance osteogenic differentiation by sponging miRNAs that inhibit β-catenin signaling, while also participating in NF-κB activation under inflammatory stress [35]. *MALAT1* was reported to modulate the differentiation of human periodontal ligament stem cells by regulating miR-155-5p [36]. A study using PDLSCs found that *MALAT1* and FGF2 mRNA were significantly upregulated in periodontitis-derived cells compared with cells derived from healthy teeth [37]. The involvement of *MALAT1* in epigenetic regulation of inflammatory processes has been demonstrated in studies of diabetic retinopathy, with increased expression of *MALAT1*, TNF-α, and IL-6 in the vitreous humor from diabetic patients [38].

Also, the overexpression of *MALAT1* in the cytoplasm of dental pulp cells in a high-glucose microenvironment was correlated with elevated levels of osteogenic and mineralization factors, including TGFβ-1, TGFβ-2, BMP2, BMP4, RUNX2, ALP, DMP-1, and dentin sialophosphoprotein (DSPP). After inhibition of *MALAT1* only TGFβ-1, BMP2, MSX2, SP7, ALP, and DSPP were significantly downregulated in DPCs [39,40]. *MALAT1* knockdown played a protective role in the LPS-induced acute lung injury rat model and inhibited the LPS-induced inflammatory response in murine alveolar macrophages in vitro [41].

In our experiments, LPS-induced variations in *MALAT1* expression level could show a context-dependent balance between reparative signaling and inflammation-driven inhibition.

*MEG3* is a tumor suppressor lncRNA with documented roles in osteogenic differentiation and inflammatory regulation. *MEG3* can inhibit NF-κB signaling and promote osteogenesis by modulating the Wnt/β-catenin and BMP pathways [42,43]. Dysregulation of *MEG3* expression following LPS exposure may thus contribute to sustained inflammatory signaling and defective osteoblast maturation.

*NEAT1* is a key organizer of nuclear paraspeckles and a potent regulator of inflammatory gene expression [44]. *NEAT1* was upregulated under LPS treatment. The lncRNA has been shown to promote NF-κB activation and cytokine production in response to bacterial LPS [45]. In mesenchymal stem cells, elevated *NEAT1* expression correlates with impaired osteogenic differentiation, likely through activation of the inflammatory pathway and suppression of osteogenic transcription factors.

Our findings support a model in which bacterial LPS disrupts the osteogenic differentiation of PL-MSCs by orchestrating the regulation of lncRNAs that connect inflammatory signaling (NF-κB) with osteogenic pathways (Wnt/β-catenin and RUNX2). Targeting these lncRNA-regulatory networks could therefore offer a new therapeutic approach to restore regenerative ability in inflammatory periodontal conditions.

We demonstrated that LPS-induced inflammation disrupts osteogenic differentiation of periodontal ligament stem cells at multiple levels, affecting matrix protein expression, mineralization, and non-coding RNA regulation. The bacterial origin was shown to be essential in shaping host cellular responses, as evidenced by distinct effects observed between *E. coli* and *P. gingivalis* LPS. These results offer new insights into the molecular mechanisms underlying bone remodeling driven by inflammation and support targeting lncRNA pathways to restore regenerative capacity in inflammatory settings.

A limitation of the present study is the use of a single, standardized strain of *P. gingivalis* LPS. In our experiments, we used ultrapure LPS from InvivoGen to ensure high reproducibility and minimize contamination from other bacterial components, providing a reliable baseline for evaluating lncRNA expression changes. However, further research comparing LPS derived from multiple clinical strains is warranted to fully capture the diversity of biological effects in the subgingival environment and their specific impact on PL-MSC osteogenic potential.

## 4. Materials and Methods

### 4.1. Periodontal Ligament Stem Cells (PL-MSC) Culture

Previously isolated mesenchymal stem cells from the periodontal ligament, kindly provided by Prof. Bosca Bianca, from the “Iuliu Hatieganu” University of Medicine and Pharmacy—Histology Department, were used. Periodontal ligament stem cells were isolated from healthy dental tissues obtained from patients indicated for extraction, after informed consent was obtained. The patients had no comorbidities and showed no infectious complications related to their dental tissues. The study was conducted in accordance with the declaration of Helsinki and approved by the Ethics Committee of “Iuliu Hatieganu” University of Medicine and Pharmacy, Cluj-Napoca (343/02.10.2014, 2 October 2014). The cells were isolated from the periodontal ligament in the middle root zone. Primary culture was obtained by mechanically processing tissue fragments and culturing cells in standard mesenchymal stem cell medium: DMEM with 4.5 g glucose/F12-HAM (1/1) + 15% FBS (Fetal Bovine Serum), 2 mM L-glutamine, 1% penicillin–streptomycin solution, 1% NEA solution (Non-Essential Amino acids), 55 µM β-mercaptoethanol, and 1 mM sodium pyruvate (all reagents from Sigma-Aldrich Chemie GmbH, Munich, Germany). The isolated cells demonstrated increased proliferation and a fibroblast-like appearance. Immunophenotyping by flow cytometry performed at the 6th passage showed variable expression levels of CD29, CD49e, CD73, CD90, CD105, and CD166. The PL-MSCs were negative for CD34, CD45, and CD117 [46].

### 4.2. Bone Differentiation of MSCs from Periodontal Ligament (PL-MSC)

To obtain osteoblastic precursors, we used a standard in vitro differentiation protocol [47]. PL-MSCs were seeded in Cole flasks in osteogenic medium: DMEM high glucose/F12-HAM with 15% FBS, 1% NEA, 1% penicillin–streptomycin, 2 mM glutamine, ascorbic acid phosphate (50 µg/mL), dexamethasone (20 µM), and β-glycerophosphate (10 mM). When the cells reached subconfluence, they were passaged and reseeded in the same culture medium.

### 4.3. Initial Experiments (Mineralization)

The LPS dose of 100 ng/mL used was indeed different for the mineralization experiments, as these were among the first experiments performed in this study. The doses were established based on the literature, such as Guo et al. [15], who observed a significant decrease in ALP, Coll1, BSP, and OCN mRNA levels even at 100 ng/mL; the effects at 1 μg/mL were quite similar. For subsequent experiments, we considered that the effects of a higher dose (1 μg/mL) would be more relevant.

#### 4.3.1. Rationale for 100 ng/mL (Initial Mineralization Experiments)

This concentration was selected to assess early or moderate inhibitory effects on mineralization. It is well supported by previous studies, such as Kadono et al. (1999), who used a range of 0–100 ng/mL to investigate osteoblastic inhibition [28], and Barksby et al. (2009) [48], who demonstrated that 100 ng/mL effectively induces immune responses in monocytes [48]. Furthermore, Guo et al. (2014) reported significant downregulation of ALP, COL1, and OCN mRNA expression at this concentration [15].

#### 4.3.2. Rationale for 1 μg/mL (Marker Expression and Comparative Analysis)

For subsequent experiments, the concentration was increased to 1 μg/mL to simulate a stronger, more clinically relevant inflammatory challenge characteristic of the subgingival environment. This dose is widely recognized as a standard in recent high-impact studies. For instance, He et al. (2025) [49] and Pedrosa et al. (2022) used 1 μg/mL to investigate the behavior of human dental pulp and periodontal ligament (PDL) stem cells under bacterial stress [50]. Similarly, Li et al. (2021) and Bozkurt et al. (2021) identified 1 μg/mL (equivalent to 1000 ng/mL) as an effective concentration for inducing pyroptosis and cytokine production in gingival fibroblasts and cementoblasts [51,52].

In addition, Santos et al. (2025) and Xing et al. (2019) validated this concentration as an upper-range dose for studying the immunomodulatory and osteogenic effects of *E. coli* LPS [53,54].

Alizarin red staining of pre-differentiated osteoblasts treated with LPS from *E. coli*.

MSCs from PL were pre-differentiated for 4 weeks in osteogenic medium and seeded at a density of 5 × 10^4^ cells per well in 12-well plates, either in 1 mL of osteogenic medium or in standard stem cell medium. After cell adhesion, they were treated with 100 ng/mL LPS from *E. coli*. Half of the wells served as controls. After 5 days, the cells were fixed and stained with alizarin red to assess mineralization. The fixed cells were treated with 4% paraformaldehyde for 20 min, washed with PBS, and then with double-distilled water. The samples were incubated with a 2% alizarin red solution at pH 4.2 for 20 min with gentle shaking on a rotary shaker. The alizarin red solution was discarded, followed by thorough washes with double-distilled water and a final wash with PBS. Microscopic images of calcium deposits were captured using a Zeiss Axiovert D1 inverted microscope with an AxioCAM MRC color camera (Carl Zeiss, Oberkochen, Germany). 

### 4.4. Expression of Osteogenesis Markers of PL-MSCs Treated with LPS_E. coli_ and LPS_Pg_

The osteogenic differentiation of PL-MSCs was induced using osteogenic medium: DMEM high glucose/F12 (1:1), 15% FCS, 1% NEA, 1% penicillin–streptomycin, 2 mM L-glutamine, ascorbic acid phosphate (50 µg/mL), dexamethasone (20 µM), and β-glycerophosphate (10 mM) [55,56,57]. PL-MSCs were seeded on chamber slides with 16 wells at a cell density of 2 × 10^4^ cells per well. Cells were treated with 1 µg/mL LPS*_E. coli_* (Invitrogen™—eBioscience™ Lipopolysaccharide Solution, 500X, Thermo Fisher Scientific, Waltham, MA, USA) and 1 µg/mL LPS*_Pg_* (LPS-PG Ultrapure, InvivoGen, San Diego, CA, USA). Controls were cultivated in standard stem cell medium and treated with LPS. After 5 days of differentiation in the presence of LPS, the cells were fixed with 4% paraformaldehyde, and immunocytochemical staining was performed using the following primary antibodies: anti-human mouse IgG1 osteopontin (Invitrogen), anti-human mouse IgG2a osteocalcin (Santa Cruz Biotechnologies, Dallas, TX, USA), anti-human goat polyclonal IgG collagen 1A (Santa Cruz Biotechnologies), anti-human mouse IgG1 SPARC (osteonectin) (Santa Cruz Biotechnologies), and anti-human mouse IgG1 dentin matrix protein-1 DMP-1 (EMD Millipore, Burlington, MA, USA) [58,59], all at a 1:50 dilution with overnight exposure at 4 °C. FITC-labeled secondary antibodies (Santa Cruz Biotechnologies) were applied to the samples at the same dilution. Nuclei were stained with a mounting medium containing DAPI. Fluorescence images were captured using a Nikon Eclipse E600 microscope (Stroombaan 14, 1181 VX Amstelveen, The Netherlands) equipped with a digital color camera. The green fluorescence intensity, represented as the normalized integrated density of captured fluorescence images, was normalized to the number of nuclei using ImageJ (v1.52P, NIH) (public domain NIH Image program developed at the U.S. National Institutes of Health https://imagej.net/nih-image/), accessed 30 March 2026.

### 4.5. Gene Expression Evaluation by RT-qPCR

For the extraction of total RNA, the TRIzol (TriReagent, Sigma-Aldrich) protocol was used, and both quantitative and qualitative controls were performed with the NanoDrop 1000 spectrophotometer (Thermo Scientific). cDNA synthesis was carried out using the High-Capacity cDNA Reverse Transcription Kit (Applied Biosystems™, Waltham, MA, USA) with 1500 ng of total RNA, following the manufacturer’s instructions. For RT-qPCR, we employed the PowerUp SYBR Green Master Mix (Applied Biosystems™) and ran the reactions on the ViiA7 instrument (Applied Biosystems™, Waltham, MA, USA) according to the manufacturer’s protocol. Five lncRNAs and three coding genes were analyzed, with B2M and GAPDH serving as housekeeping genes (Table 2). Changes in the expression levels of the studied lncRNAs and genes were assessed using the ΔΔCT method based on the CT values obtained. The same was done for the genes in Table 3. 

### 4.6. Statistical Analysis

Standard deviation (Mean ± SD) and a t-test were used to evaluate differences between the experimental and control conditions (*p*-value < 0.05 was considered statistically significant). Bar charts with error bars representing standard deviations were used to describe gene expression, by group, differentiation, and time. T-tests were used to compare groups for gene expression in the univariate analyses. To isolate the independent effects of the groups, differentiation, and time on gene expression, multiple linear regression models were fit with the gene expression as dependent variables, and groups, differentiation, and time as independent variables. For all multiple linear regression models, standard assumptions were examined. Normality of residuals was examined with Q–Q plots, whereas heteroskedasticity was checked with scale-location plots and formally with the Breusch–Pagan test. Multicollinearity was checked with generalized variance inflation factors. Linearity of the functional form between the response variable and each predictor was checked with component plus residual (partial residual) plots. To treat potential heteroskedasticity, robust standard errors were calculated with heteroskedasticity-consistent (HC3) sandwich estimators. Where residuals were nonnormal, Box–Cox transformations were implemented upon the dependent variable to enhance the residuals’ distributional characteristics. The model coefficients, along with 95% confidence intervals and *p*-values were presented. Effects plots were reported to present the independent effect of each variable on gene expression. Statistical analyses and graphical representations were performed using the R environment for statistical computing and graphics (R Foundation for Statistical Computing, Vienna, Austria), version 4.3.2.

## 5. Conclusions

The focus of our study was to determine the expression levels of key lncRNAs associated with inflammation-related bone remodeling in response to different bacterial stimuli.

We found decreased expression of *GAS5*, *MEG3*, and *MALAT1* in the LPS vs. CTR comparison, decreased COL1A1 in LPS-PG vs. CTR, and increased OSTEOPONTIN in LPS vs. CTR. Differentiation was significantly associated with reduced expression of *XIST* and *NEAT1*. Time exerted significant effects on *GAS5*, *MEG3*, *XIST*, and *MALAT1*, with lower expression at 48 h compared with 6 h, and on COL1A1, which was significantly reduced at both 24 h and 48 h relative to 6 h.

Various bacterial lipopolysaccharides interfere with the osteogenic differentiation of periodontal ligament mesenchymal stem cells, leading to decreased extracellular matrix mineralization and altered expression of essential osteogenic markers. Additionally, the inhibitory effects of LPS on osteogenesis depend on the pathogen, with *E. coli* LPS having a stronger suppressive effect on matrix mineralization than *Porphyromonas gingivalis* LPS, emphasizing the importance of microbial specificity in shaping host bone responses. Inflammatory stimulation changes extracellular matrix gene expression, particularly affecting collagen type 1 and osteopontin, indicating a shift from effective osteogenic maturation toward an inflammatory matrix-remodeling phenotype. Osteocalcin expression, a late marker of osteoblast maturation, was absent or severely reduced, as terminal osteogenic differentiation is hindered under inflammatory conditions. 

Long non-coding RNAs (*GAS5*, *MALAT1*, *MEG3*, and *NEAT1*) show coordinated regulation in response to LPS exposure, supporting their role as key regulatory integrators of inflammatory and differentiation signals. Multivariate computational analyses reveal coherent transcriptional programs, demonstrating that inflammation-induced osteogenic dysregulation involves coordinated gene networks rather than isolated gene-specific changes. LncRNAs could function as regulatory modules and as extracellular matrix genes, representing downstream outputs of inflammatory reprograming.

Our findings deepen the mechanistic understanding of inflammation-related bone remodeling and suggest that targeting lncRNA-centered regulatory networks could be a promising way to restore osteogenic potential in inflammatory settings. It may also prove a promising strategy for enhancing periodontal regeneration and ensuring optimal results in orthodontic therapy.

## Figures and Tables

**Table 1:** Gene expression was predicted with a multiple linear regression based on group, differentiation, and time.

Gene	Variable	Coefficient (95% CI)	*p* Value
*GAS5*	Group (LPS vs. CTR)	−0.43 (−0.6; −0.25)	<0.0001 *
	Group (LPS-PG vs. CTR)	−0.24 (−0.5; 0.01)	0.0633
	LP (LPdiff vs. LP)	−0.17 (−0.36; 0.02)	0.0742
	Time (h), (24 vs. 6)	−0.18 (−0.41; 0.05)	0.1251
	Time (h), (48 vs. 6)	−0.25 (−0.48; −0.02)	0.0330 *
*MEG3*	Group (LPS vs. CTR)	−0.29 (−0.55; −0.02)	0.0363 *
	Group (LPS-PG vs. CTR)	−0.13 (−0.47; 0.21)	0.438
	LP (LPdiff vs. LP)	0.11 (−0.17; 0.4)	0.4222
	Time (h), (24 vs. 6)	−0.03 (−0.39; 0.32)	0.8473
	Time (h), (48 vs. 6)	−0.34 (−0.58; −0.1)	0.0070 *
*XIST*	Group (LPS vs. CTR)	0.14 (−0.45; 0.72)	0.6322
	Group (LPS-PG vs. CTR)	0.26 (−0.54; 1.06)	0.5166
	LP (LPdiff vs. LP)	−0.74 (−1.29; −0.19)	0.0104 *
	Time (h), (24 vs. 6)	−0.24 (−0.91; 0.43)	0.4769
	Time (h), (48 vs. 6)	−0.97 (−1.6; −0.35)	0.0031 *
*NEAT1*	Group (LPS vs. CTR)	0.02 (−0.38; 0.41)	0.9352
	Group (LPS-PG vs. CTR)	0.22 (−0.22; 0.66)	0.3201
	LP (LPdiff vs. LP)	−0.63 (−0.94; −0.32)	0.0002 *
	Time (h), (24 vs. 6)	−0.18 (−0.53; 0.17)	0.2969
	Time (h), (48 vs. 6)	0.38 (0; 0.76)	0.0512
*MALAT1*	Group (LPS vs. CTR)	−0.67 (−1.2; −0.15)	0.0136 *
	Group (LPS-PG vs. CTR)	−0.47 (−1.06; 0.12)	0.1129
	LP (LPdiff vs. LP)	0.42 (−0.08; 0.91)	0.0944
	Time (h), (24 vs. 6)	−0.16 (−0.81; 0.5)	0.6314
	Time (h), (48 vs. 6)	1.37 (0.68; 2.06)	0.0002 *
OSTEOPONTIN	Group (LPS vs. CTR)	1.13 (0.18; 2.08)	0.0217 *
	Group (LPS-PG vs. CTR)	0.68 (−0.45; 1.81)	0.2296
	LP (LPdiff vs. LP)	−0.46 (−1.43; 0.5)	0.3369
	Time (h), (24 vs. 6)	−0.39 (−1.6; 0.82)	0.5139
	Time (h), (48 vs. 6)	0.18 (−1.05; 1.4)	0.7685
OSTEOCALCIN	Group (LPS vs. CTR)	−0.72 (−1.92; 0.47)	0.2251
	Group (LPS-PG vs. CTR)	−0.57 (−2.71; 1.57)	0.5921
	LP (LPdiff vs. LP)	−1.6 (−3.3; 0.1)	0.0648
	Time (h), (24 vs. 6)	1.04 (−1.3; 3.38)	0.3738
	Time (h), (48 vs. 6)	0.79 (−0.33; 1.91)	0.1598
COLLAGEN 1	Group (LPS vs. CTR)	−0.13 (−0.29; 0.04)	0.1202
	Group (LPS-PG vs. CTR)	−0.2 (−0.38; −0.02)	0.0328 *
	LP (LPdiff vs. LP)	−0.04 (−0.18; 0.11)	0.6193
	Time (h), (24 vs. 6)	0.08 (−0.11; 0.28)	0.3954
	Time (h), (48 vs. 6)	0.25 (0.09; 0.42)	0.0039 *

CI, confidence interval. *p*-values from tests comparing each sample with the corresponding control group; * statistically significant differences (*p* < 0.05).

**Table 2:** LncRNA and housekeeping gene sequences used in the study.

Name	Sequences
*B2M*	FW-CACCCCCACTGAAAAAGATGAG/RW-CCTCCATGATGCTGCTTACATG
*GAPDH*	FW-AGAACATCATCCCTGCCTCTAC/RW-CTGTTGAAGTCAGAGGAGACCA
*GAS5*	FW-TCGACTCCTGTGAGGTATGGT/RW-TGGGGACACAACTGTCCAT
*MEG3*	FW-GGGTCTCTCCTCAGGGATG/RW-ATGGAGAGGAGGTGGTCCTT
*XIST*	FW-TTGGCCCAGGCTCGAGT/RW-CGGGGCTCACGCCCATAA
*NEAT1*	FW-GAGAAAAGTCCAAAAGGAGCAC/RW-GGATGAGGCCTGGTCTTGT
*MALAT1*	FW-TGTCCTTATAGGCTGGCCATT/RW-AACTGCAGAGTTTGAGTGGTTTT

**Table 3:** Gene sequences investigated in the study.

Name	Sequences
OSTEOPONTIN	FW-CAGTGACCACTTCATCAGATTCATC/RW-CTAGGCATCACCTGTGCCATACC
OSTEOCALCIN	FW-ATGAGAGCCCTCAGACTCCT/RW-CAAGGGGAAGAGGAAAGAAG
COLLAGEN 1	FW-GGGATTCCCTGGACCTAAAC/RW-GGAACACCTCGCTCTCCA

## Data Availability

The data presented in this study are available on request from the corresponding author.

## Abbreviations

The following abbreviations are used in this manuscript:

PL-MSCs	Periodontal ligament mesenchymal stem cells
PDLSCs	Periodontal ligament stem cells
LPS	Lipopolysaccharides
*E. coli*	*Escherichia coli*
*Pg*	*Porphyromonas gingivalis*
lncRNAs	Long non-coding RNAs
DMP-1	Dentin matrix protein-1
*GAS5*	Growth arrest-specific transcript 5
*MALAT1*	Metastasis Associated Lung Adenocarcinoma Transcript 1
*MEG3*	Maternally expressed gene 3
*NEAT1*	Nuclear Enriched Abundant Transcript 1
*XIST*	X-inactive specific transcript
CTR	Control
COL1A1	Collagen Type 1
FBS	Fetal bovine serum
NEA	Non-essential amino acids
OS	Osteogenic medium
OPN	Osteopontin
OC	Osteocalcin
ON	Osteonectin
CI	Confidence interval
TLR	Toll-like receptor
ALP	Alkaline phosphatase
BMP	Bone morphogenetic protein
TGF	Transforming growth factor
DSPP	sialo phosphoprotein
